# New Item Selection Method Accommodating Practical Constraints in Cognitive Diagnostic Computerized Adaptive Testing: Maximum Deviation and Maximum Limitation Global Discrimination Indexes

**DOI:** 10.3389/fpsyg.2021.619771

**Published:** 2021-05-17

**Authors:** Junjie Li, Lihua Ma, Pingfei Zeng, Chunhua Kang

**Affiliations:** Key Laboratory of Intelligent Education Technology and Application of Zhejiang Province, Zhejiang Normal University, Jinhua, China

**Keywords:** balance attribute coverage, cognitive diagnostic computerized adaptive testing, attribute discrimination index, item exposure control, mastery pattern correct classification rate

## Abstract

Maximum deviation global discrimination index (MDGDI) is a new item selection method for cognitive diagnostic computerized adaptive testing that allows for attribute coverage balance. We developed the maximum limitation global discrimination index (MLGDI) from MDGDI, which allows for both attribute coverage balance and item exposure control. MLGDI can realize the attribute coverage balance and exposure control of the item. Our simulation study aimed to evaluate the performance of our new method against maximum global discrimination index (GDI), modified maximum GDI (MMGDI), standardized weighted deviation GDI (SWDGDI), and constraint progressive with SWDGDI (CP_SWDGDI). The results indicated that (1a) under the condition of realizing the attribute coverage balance, MDGDI had the highest attribute classification accuracy; (1b) when the selection strategy accommodated the practical constraints of the attribute coverage balance and item exposure control, MLGDI had the highest attribute classification accuracy; (2) adding the item exposure control mechanism to the item selection method reduces the classification accuracy of the attributes of the item selection method; and (3) compared with GDI, MMGDI, SWDGDI, CP_SWDGDI, and MDGDI, MLGDI can better achieve the attribute-coverage requirement, control item exposure rate, and attribute correct classification rate.

## Introduction

Cognitive diagnostic assessment (CDA) is a recently popular assessment method in theoretical studies on psychological testing. CDA was developed to measure cognitive skills (Leighton and Gierl, 2007; Gierl et al., 2008). When based on the classical test theory (CTT), CDA provides examinee scores. When based on the multidimensional item response theory, CDA provides multidimensional ability scores, which details the advantages and disadvantages of the examinee in a given content domain, aiding the assessment of the examinees by administrators (Yao and Boughton, 2007; Lee et al., 2012).

Interest in cognitive diagnosis is largely motivated by the need for formative assessments. Computerized adaptive testing (CAT) combines test theory with computer technology to improve testing efficiency (Weiss, 1982), which has become a promising method in psychological and educational measurement. In addition, items in CAT have been executed in examinations for items that have matched the estimating ability for candidates (Mao and Xin, 2013; Chang, 2015). Recently, to maximize the benefits of both CDA and CAT, researchers have attempted to combine CDA with CAT and named it cognitive diagnostic CAT (CD-CAT) (Xu et al., 2003; McGlohen and Chang, 2008; Cheng, 2009a, b). CD-CAT, which has the characteristics of a tailor-made test, is promising and will be influential in future educational practices. CD-CAT has received an increasing scholarly attention worldwide (Kang et al., 2017; Huebner et al., 2018).

The goal of CAT is to conduct individualized item selection tests based on the most currently estimated ability of the participant; thus, the determination of an optimal item selection method is key in CAT. Although many item selection strategies have been constructed in the item response theory–based CAT, few applicable item selection strategies are currently available in CD-CAT. Therefore, this study aimed to construct a selection strategy that is suitable for CD-CAT. Based on the difference distribution criteria of the potential attribute-mastery pattern at the item level, researchers have proposed a selection criteria, such as the Kullback–Leibler (KL)-based global discrimination index (GDI), Shannon entropy procedure (Xu et al., 2003), and the posterior-weighted KL information (PWKL; Cheng, 2009a, b). However, the aforementioned item selection methods focus on the maximum information of the item without considering the attribute coverage balance of the test and exposure control of the item. Therefore, the aforementioned item selection methods face the following two problems. First, attribute coverage imbalance may cause the test results to be unreliable. Cheng (2010) also pointed out that it is of great importance to ensure that each attribute in the test has been measured adequately or the reliability of the test will be reduced. Second, an unevenly applied item bank will result in the following two situations: (1) some items increase their exposure rate in a different test, which endangers the security of the item bank, and (2) if some items are applied adequately, the item bank is poorly utilized and resources (including labor) are wasted. Although CD-CAT is increasingly used in the classroom, test security is not fundamental to the CD-CAT practices, whereas security and property balance are critical to CD-CAT developers. Specifically, the item bank must be secured because CD-CAT is a complex and expensive project. As for every item written for CD-CAT, it must be based on a complex blueprint of cognitive requirements. In addition, when specific items are used for each test, practice or memory effects may produce invalid diagnostic information for candidates who has taken the test repeatedly. Improving the utilization rate of an item bank also constitutes a research problem for the practical application of CD-CAT.

To balance the attribute coverage in CD-CAT, Cheng (2010) developed the modified maximum global discrimination index (MMGDI) to build the item selection method using the number of items that measure each attribute as the MMGDI did. The MMGDI method is based on the global discrimination index (GDI) developed by Xu et al. (2003). Although the MMGDI method achieves the balance in attribute coverage and improves the accuracy of the attribute-mastery pattern, MMGDI does not consider the exposure rate of items, which leads the MMGDI to repeatedly select some items in different tests. Lin and Chang (2018) proposed a method, the constraint progressive with standardized weighted deviation GDI (CP_SWDGDI), which allows for attribute coverage balance and exposure control (named considering the attribute balancing and exposure control). Although CP_SWDGDI considers both the attribute coverage balance and exposure control, the CP_SWDGDI selection method fails under some conditions, such as when the attribute coverage balance is satisfied.

The objective of this article is to propose a maximum deviation (MD) index and a maximum limit (ML) index, and combine them with GDI for use in CD-CAT. We first developed an item selection method MDGDI, which can achieve attribute coverage balance. Subsequently, we added an exposure control mechanism based on MDGDI and developed a CD-CAT item selection method MLGDI, that can achieve attribute coverage balance and items exposure control. The rest of this paper is organized as follows.

First, we discuss the CDM used in this study and introduce the four existing item selection algorithms for CD-CAT. Next, we introduce the MDGDI and MLGDI methods. We then evaluate MDGDI and MLGDI against the existing item selection algorithms via two simulation studies. Finally, we discuss the consequences of the simulation results and provide suggestions for further research.

## Reduced Reparameterized Unified Model

The reduced reparameterized unified model (RRUM) is used in the current study (Hartz, 2002; Hartz and Roussos, 2008), because previous studies have demonstrated that the RRUM is very useful for formative assessment in practice (Wang et al., 2011). The item response function of the RRUM is defined as,

p(Yij=1|ai)=πj*∏k∈Ajrjk(1-aik)*qjk

where, $0<\pi_{j}^{{}^{*}}<1$ is the probability of a correct answer for an examinee who has mastered all the attributes required for item *j*, and $0<r_{jk}^{{}^{*}}<1$ is a penalty parameter that reduces the probability of a correct response by a factor of $r_{jk}^{{}^{*}}$ for examinees who do not possess attribute *k*.

## Existing Item Selection Methods

### Global Discrimination Index (GDI)

The KL information was first introduced to CAT research in Chang and Ying’s (1996) groundbreaking paper on global information. The KL information has since been applied to various studies on CAT. For example, CAT was established based on a non-parametric item response theoretical model (Xu and Douglas, 2006), and CAT has been applied to classification (Weissman, 2007) and cognitive diagnostic applications (McGlohen and Chang, 2008; Cheng, 2009a, b). The KL information, which measures the distance or divergence between two probability distributions *f*(*x*) and *g*(*x*) (Cover and Thomas, 1991; Kaplan et al., 2015), is defined as follows:

$$d\left[f,g\right]=E_{f}\left[\log\left[\frac{f\left(x\right)}{g\left(x\right)}\right]\right]$$

In CD-CAT, information refers to the ability of an item to distinguish between a pair of attribute patterns. In this sense, KL information in diagnostic classification reflects the distance between two conditional distributions, that is, $f\left(X_{ij}|{\hat{a}}_{i}\right)$ is the distribution on the currently estimated attribute under condition *X*_*ij*_, and *f*(*X*_*ij*_|*a*_*t*_) is the distribution in the real state under condition *X*_*ij*_. This logic gives the KL equation of CD-CAT:

$$KL_{j}\left({\hat{a}}_{i}||a_{t}\right)=\sum_{x=0}^{1}\log\left(\frac{p\left(X_{ij}=x|{\hat{a}}_{i}\right)}{p\left(X_{ij}=x|a_{t}\right)}\right)p\left(X_{ij}=x|{\hat{a}}_{i}\right)$$

Xu et al. (2003), who considered that the true potential is unknown and that 2^*k*^ possible states exist, proposed the GDI with the following formula:

GDIj(a^i)=∑c=12k[∑x=01log(p(Xij=x|a^i)p(Xij=x|a^)c)p(Xij=x|a^i)]

This index is the sum of the KL distances between p(X|ija^i) and all possible potential states $p\left(X_{ij}|{\hat{a}}_{c}\right)$. Items with large GDI values have a correspondingly high recognition between the estimating attribute patterns and all other possible cognitive profiles. An item with a maximum GDI (MGDI) will be administered as the next item for a specific examinee. In Xu et al. (2003), the MGDI method exhibited a good performance in restoring the pattern of student attribute mastery.

### The Maximum Modified Global Discrimination Index (MMGDI)

The disadvantage of the GDI approach is that it does not consider property balancing or exposure control. Cheng and Chang (2009) introduced the maximum priority index (MPI) method for the selection of items that satisfy the constraints in the IRT-based CAT. In a subsequent study, Cheng (2010) extended the MPI method to CD-CAT to achieve balance attribute override. The attribute coverage balance index (ABI) is defined as follows:

$$ABI_{j}=\prod_{k=1}^{K}{\left(\frac{B_{k}-b_{k}}{B_{k}}\right)}^{q_{jk}}$$

where, *B*_*k*_ is the lower bound of the number of items required to measure attribute *k*, *b*_*k*_ is the number of items measuring attribute *k* that has already been selected, and *q*_*jk*_ is the element of the Q matrix. Cheng (2010) added the ABI to GDI and constructed the MMGDI item selection method, which is defined as follows:

$$MMGDI_{j}\left({\hat{a}}_{i}\right)=\prod_{k=1}^{K}{\left(\frac{B_{k}-b_{k}}{B_{k}}\right)}^{q_{jk}}.GDI\left({\hat{a}}_{i}\right)$$

Modified maximum GDI makes a GDI-based strategy more precise. Specifically, MMGDI attributes in the balance tends only toward the choice of measurement index in the selected item of a single attribute (Mao and Xin, 2013), and, in ABI, there may be situations where negative and negative multiply to be positive, which affects the efficiency of the item selection method.

### The Standardized Weighted Deviation GDI Method (SWDGDI)

Lin and Chang (2018) proposed a new attribute-balancing item selection criterion, namely the Weighted Deviation GDI (WDGDI), which multiplies GDI by the Weighted Deviation Index (WD). To place the WD and the GDI metrics on an equal footing, they standardized the WD and GDI values and named it the standardized WDGDI (SWDGDI). The SWDGDI method is defined as follows:

$$\begin{array}{c} SWDGDI_{j}\left({\hat{a}}_{i}\right)=\left(\frac{Max\left(WD_{j}\right)-WD_{j}}{Max\left(WD_{j}\right)-Min\left(WD_{j}\right)}\right)\times \\ \;\left(\frac{GDI_{j}\left({\hat{a}}_{i}\right)-Min\left(GDI_{j}\left({\hat{a}}_{i}\right)\right)}{Max\left(GDI_{j}\left({\hat{a}}_{i}\right)\right)-Min\left(GDI_{j}\left({\hat{a}}_{i}\right)\right)}\right) \end{array}$$

$$WD_{j}=\sum_{k=1}^{K}\left(W_{k}D_{jL_{k}}\right)+\sum_{k=1}^{K}\left(W_{k}D_{jU_{k}}\right)$$

$$D_{jL_{k}}=\left\{ \begin{array}{c} L_{k}-q_{k},q_{k}<L_{k}\\ 0,q_{k}\geq L_{k} \end{array}\right.\;D_{jU_{k}}=\left\{ \begin{array}{c} q_{k}-U_{k},q_{k}\geq U_{k}\\ 0,q_{k}<U_{k} \end{array}\right.$$

where, *W*_*k*_ is the weight for the *k*^*th*^ attribute, and *D*_*jLk*_ and *D*_*jUk*_ correspond to the positive deviations from the minimal (i.e., lower boundary) and maximal (i.e., upper boundary) numbers, respectively, of the items required to assess the *k*^*th*^ attribute when item *j* is included in the test. For each constraint k, *D*_*jLk*_ is defined as (*L*_*k*_−*q*_*k*_) and *D*_*jUk*_ is defined as (*U*_*k*_−*q*_*k*_), where *L*_*k*_ and *U*_*k*_, respectively, denote the lower and upper bounds for the *k*^*th*^ attribute constraint. The term *q*_*k*_ represents the expected number of items measuring the *k*^*th*^ attribute that would have been obtained if item candidate *j* was included in the test.

With the attribute balancing considered, the largest SWDGDI item is selected first in the test rather than the GDI’s largest project.

### The Constraint Progressive With SWDGDI (CP_SWDGDI)

In order to balance the attribute coverage and control the item exposure rate, Lin and Chang (2018) adopted a progressive exposure control algorithm in SWDGDI. The Constrained Progressive Algorithm is described as follows:

$$P\_INFO_{j}=\left(1-\frac{X}{L}\right)R_{j}+\frac{X}{L}\times R_{jI}$$

$$R_{j}=U\left(0,\max\left(\mathrm{information}\right)\right)\;R_{jI}=U\left(LB_{j},UB_{j}\right)$$

$$\begin{array}{c} LB_{j}=Info_{j}-\left(Info_{j}-Min\right)/s\;UB_{j}=Info_{j}\\ \;+\left(Max-Info_{j}\right)/s \end{array}$$

In the progressive exposure control algorithm constructed by Lin and Chang (2018), the adjustment information interval parameter *s* was added. However, with regard to practical applications, Lin and Chang (2018) offered no specific suggestions for determining the value of *s*. Therefore, the appropriate value of *s* may differ between the conditions and number of attributes, which makes determining the value of *s* difficult in practical applications.

When replacing *Info*_*j*_ with *SWDGDI*_*j*_, the CP_SWDGDI became:

$$\begin{array}{c} CP\_SWDGDI_{j}\left({\hat{a}}_{i}\right)=\frac{r\_\max}{r_{j}}\\ \times \left[\left(1-\frac{X}{L}\right)R_{j}+\frac{X}{L}\times R_{jI}\right]R_{jI}=U\left(LB_{j},UB_{j}\right) \end{array}$$

$$LB_{j}=SWDGDI_{j}\left({\hat{a}}_{i}\right)-\left(SWDGDI_{j}\left({\hat{a}}_{i}\right)-Min\right)/s$$

$$UB_{j}=SWDGDI_{j}\left({\hat{a}}_{i}\right)+\left(Max-SWDGDI_{j}\left({\hat{a}}_{i}\right)\right)/s$$

where, *r_max* is the maximum exposure rate for the title and *r*_*j*_ is the current exposure rate for the item.

## Proposed Item Selection Methods

### Maximum Deviation Index With GDI (MDGDI)

In order to make all the attributes relatively balanced throughout the test and to reduce the tendency of the selection strategy to choose certain types of items more often due to the index added, the maximum deviation index (MD) was developed. MD limits the difference between the maximum and minimum measurement times of an attribute within a certain range. The definition of MD is as follows:

MD=j{1,max⁡(qjk)-min⁡(qjk)≤LB0,max⁡(qjk)-min⁡(qjk)>LB

where, *LB* is the lowest number of attributes, *q*_*jk*_ is the number of attributes to be investigated if the next item is *j*, and *MD* is the deviation index.

Now the maximum deviation global discrimination index (MDGDI) becomes:

$$MDGDI_{j}=MD_{j}\times GDI{}_{j}$$

The item that yields the largest MDGDI is offered for a specific examinee as the next item.

### Combining MD Index and Limited Exposure Control Index With GDI (MLGDI)

Although CP_SWDGDI considers both the attribute coverage balance and exposure control in the selection strategy, for the exposure control part of CP_SWDGDI, the variable*s*must be established by the manager themself, and the appropriate *s* value may differ under different conditions, which makes it difficult to determine the value of *s*.

#### Limiting Exposure Index

In this study, we proposed a limiting exposure index to control the exposure rate of items. The idea of limiting exposure index was built upon with the aims of (1) eliminating the need to determine the crucial parameter *s* in the random part and (2) making the exposure index more concise. The limit exposure index is comprised of two parts: the random part and the limit maximum exposure. The random part is based on the idea of asymptotic behavior, and the amount of information in item *J* after increasing the random part is expressed as *RI*_*j*_, where *RI*_*j*_ = *U*(*LI*_*j*_, *UI*_*j*_) and *RI*_*j*_ is generated randomly from the uniform distribution *U*(*LI*_*j*_, *UI*_*j*_).

$$LI_{j}=GDI_{j}-\left(x/L\right)\times \left(GDI_{j}-\min\left(GDI\right)\right)$$

$$UI_{j}=GDI_{j}+\left(x/L\right)\times \left(\max\left(GDI\right)-GDI_{j}\right)$$

where, GDI is the GDI information of the remaining items in the item bank; *x* is the current test length; *L* is the maximum test length; and *LI*_*j*_ and *UI*_*j*_ are the lower bound and upper line of *U*(*LI*_*j*_, *UI*_*j*_), respectively. As the length of the test increases, *LI*_*j*_ and *UI*_*j*_ approach the original *GDI*_*j*_, and the random *RI*_*j*_ approaches *GDI*_*j*_. Therefore, the information of the items becomes more accurate.

In addition, the component that limits the maximum exposure rate is as follows,

$$Lr_{j}\left\{ \begin{array}{c} 1,r_{j}<r\\ 0,r_{j}\geq r \end{array}\right.$$

where, *r* is the maximum exposure rate and *r*_*j*_ is the current exposure rate of item *j*. If the exposure rate *r*_*j*_ of the next problem *j* is greater than or equal to the maximum exposure rate *r* of the problem, then *Lr*_*j*_ = 0; if *r*_*j*_ is less than *r*, then *Lr*_*j*_ = 1.

To maximize the participant’s exposure rate restrictions, the GDI item selection method was applied with the limited exposure index as follows:

$$LGDI_{j}=Lr_{j}\times RI_{j}$$

#### Combining MD Index and Limited Exposure Control Index

According to the aforementioned MD index and limited exposure index, this study proposed the maximum limitation global discrimination indexes that considers both the attribute coverage balance and exposure control as follows:

$$MLGDI_{j}=MD_{j}\times LGDI_{j}$$

During the MLGDI procedure, an item with the maximum MLGDI value will be selected for administration.

## Simulation Study

### Study I

Study I is a simulation conducted to investigate the performance of MDGDI against GDI, MMGDI, and SWDGDI.

#### Item Pools

Item pools were constructed based on the study of Wang et al. (2020). Three item pools were designed in this study, denoted as the low discrimination (LD), high discrimination (HD), and hybrid discrimination (HyD) item pools, respectively. Each item pool contained 775 items and measured five attributes in total (Wang et al., 2011; Huebner et al., 2018). In the LD item pool, item parameters $\pi_{j}^{{}^{*}}$ and $r_{jk}^{{}^{*}}$ were generated from uniform distributions U(0.75, 0.95) and U(0.15, 0.50), respectively. In the HD item pool, $\pi_{j}^{{}^{*}}$ and $r_{jk}^{{}^{*}}$ were generated from uniform distributions U(0.75, 0.95) and U(0.05, 0.40), respectively. In the HyD item pool, $\pi_{j}^{{}^{*}}$ and $r_{jk}^{{}^{*}}$ were generated from uniform distributions U(0.75, 0.95) and U(0.05, 0.50), respectively. Table 1 represents the descriptive statistics of item parameters of LD item pool, HD item pool, and HyD item pool.

**TABLE 1:** Descriptive statistics of item parameters of the LD, HD, and HyD item pools.

		$\pi_{j}^{*}$	$r_{1}^{*}$	$r_{2}^{*}$	$r_{3}^{*}$	$r_{4}^{*}$	$r_{5}^{*}$
LD item pool	Min	0.750	0.151	0.152	0.152	0.151	0.151
	Mean	0.850	0.327	0.323	0.325	0.326	0.324
	Max	0.950	0.500	0.499	0.499	0.499	0.497
	SD	0.058	0.100	0.101	0.103	0.100	0.100
HD item pool	Min	0.750	0.051	0.050	0.052	0.051	0.052
	Mean	0.850	0.229	0.224	0.230	0.226	0.225
	Max	0.950	0.399	0.399	0.398	0.399	0.397
	SD	0.058	0.101	0.102	0.100	0.102	0.101
HyD item pool	Min	0.750	0.051	0.052	0.052	0.053	0.050
	Mean	0.849	0.273	0.268	0.279	0.275	0.275
	Max	0.950	0.499	0.499	0.497	0.499	0.499
	SD	0.057	0.127	0.133	0.129	0.129	0.135

*LD item pool, low discrimination item pool; HD item pool, high discrimination item pool; HyD item pool, hybrid discrimination item pool.*

#### Examinee Populations

Three examinee populations were generated, each containing 3,200 examinees. The first population (denote as Unif) assumed that the Attribute Mastery Pattern (AMP) of each examinee was generated from the uniform distribution of 32 possible pattern profiles with a probability of 1/32. In this way, each AMP had 100 examinees; meanwhile, each examinee had a 0.5 chance to master each attribute. Considering that correlations among attributes is common in practice, a multivariate normal distribution was used to describe the relationship among attributes for the second and third populations (denote as Norm). In these two groups, the mastery probabilities for the five attributes are defined as 0.45, 0.50, 0.55, 0.60, and 0.65, respectively. The correlations between attributes were set at 0.5 for the second population (low correlation) and 0.8 for the third population (high correlation). Table 2 represents the frequencies of examinees who possess each possible number of attributes.

**TABLE 2:** Frequencies of examinees exhibiting each possible number of attributes in each population.

Number of attributes		0	1	2	3	4	5
Number of examinees	Unif	100	495	999	1,005	494	107
	Norm-0.5	208	338	400	541	691	1,022
	Norm-0.8	486	270	265	301	431	1,447

#### Constraints of Attribute-Balance Coverage

The minimum measurement time of each attribute was *B*_*k*_ = 3. The s parameter in CP_SWDGDI was 1.6, r_max = 0.2 and LB = 3.

We generated a total of 27 conditions in this study (3 item pools × 3 examinee populations × 3 item selection methods). We fixed the number of items in the test to have 20 in all conditions. The first item was selected randomly from the item pool, with a maximum *a posteriori* (MAP) method used to estimate the examinee’s AMP, and the prior information of AMP assumed to follow a uniform distribution. The study procedures were implemented by the R software.

#### Evaluation Criteria

We evaluated the methods with respect to six criteria: attribute correct classification rate (ACCR), average marginal match rate (AAMR), mastery pattern correct classification rate (PCCR), item-bank exposure rate χ^2^, test overlap rate (TOR), and maximum exposure rate. The computation of the first five criteria is as follows:

$$ACCR_{k}=\sum_{i=1}^{N}I\left({\hat{a}}_{ik}=a_{ik}\right)/N$$

$$AAMR=\sum_{k=1}^{K}\sum_{i=1}^{N}I\left({\hat{a}}_{ik}=a_{ik}\right)/\left(N^{*}K\right)$$

$$PCCR=\sum_{i=1}^{N}I\left({\hat{a}}_{i}=a_{i}\right)/N$$

$$TOR=\frac{N\times \sum_{j=1}^{N_{item}}er_{j}^{2}}{\left(N-1\right)\times L}-\frac{1}{N-1}$$

$$\chi^{2}=\sum_{j=1}^{N_{item}}{\left(er_{j}-J/N_{item}\right)}^{2}/\left(J/N_{item}\right)$$

where, ${\hat{\alpha }}_{i}$ and *a*_*i*_ are the real and estimated values, respectively, of the attribute of participant *i* mastering the pattern, I(…) is an indicator function. A higher ACCR and AMMR value indicate a more accurate estimate of each participant attribute. A higher PCCR value indicates a more accurate estimate of the participant’s overall knowledge status; *er*_*j*_ is the exposure rate of item *j*, *N*_*item*_ is the size of the item bank, χ^2^ is the exposure rate index of an item, and TOR is the overlapping rate index of the test. The smaller the values of χ^2^ and TOR are, the more fully and uniformly the item strategy utilizes the item bank.

#### Results

Table 3 compares the recovery rate of each attribute and of the entire profile obtained from the four item selection methods (GDI, MMGDI, SWDGDI, and MDGDI). Clearly, the MMGDI, SWDGDI, and MDGDI methods outperformed the GDI method especially in the entire pattern recovery rate. This was because recovering the entire profile requires correctly recovering every attribute and gain the attribute level aggregates. This is in line with Cheng (2010) and Lin and Chang (2018). Among the four methods, the MDGDI method was superior. Besides, all of the methods performed best in the HD item pool, followed by the HyD item pool, and the LD item pool was the worst.

**TABLE 3:** Accuracy of the attribute classification for five attributes.

	Item selection method	Attribute (ACCR)					AAMR	PCCR
		A1	A2	A3	A4	A5		
HD-unif	GDI	0.910	0.814	0.975	0.949	0.956	0.921	0.642
	MMGDI	0.968	0.964	0.965	0.976	0.968	0.968	0.853
	SWDGDI	0.937	0.867	0.969	0.945	0.959	0.935	0.688
	MDGDI	0.990	0.984	0.992	0.991	0.990	0.989	0.953
HD-norm-0.5	GDI	0.946	0.854	0.856	0.901	0.942	0.900	0.598
	MMGDI	0.978	0.969	0.976	0.973	0.982	0.976	0.887
	SWDGDI	0.962	0.944	0.948	0.944	0.967	0.953	0.778
	MDGDI	0.991	0.988	0.991	0.991	0.995	0.991	0.959
HD-norm-0.8	GDI	0.833	0.871	0.882	0.939	0.907	0.886	0.592
	MMGDI	0.967	0.971	0.964	0.980	0.970	0.971	0.864
	SWDGDI	0.947	0.957	0.952	0.967	0.965	0.958	0.800
	MDGDI	0.989	0.994	0.991	0.993	0.990	0.991	0.962
LD-unif	GDI	0.973	0.852	0.982	0.913	0.821	0.908	0.599
	MMGDI	0.955	0.946	0.964	0.937	0.939	0.948	0.769
	SWDGDI	0.964	0.921	0.972	0.922	0.901	0.936	0.728
	MDGDI	0.972	0.963	0.978	0.960	0.960	0.967	0.868
LD-norm-0.5	GDI	0.866	0.914	0.914	0.950	0.793	0.887	0.541
	MMGDI	0.964	0.959	0.959	0.960	0.950	0.958	0.815
	SWDGDI	0.928	0.944	0.949	0.953	0.908	0.936	0.715
	MDGDI	0.977	0.975	0.981	0.985	0.967	0.977	0.900
LD-norm-0.8	GDI	0.912	0.866	0.885	0.913	0.935	0.902	0.595
	MMGDI	0.958	0.960	0.958	0.954	0.973	0.961	0.819
	SWDGDI	0.941	0.933	0.932	0.929	0.954	0.938	0.709
	MDGDI	0.965	0.970	0.974	0.964	0.985	0.972	0.877
HyD-unif	GDI	0.937	0.908	0.983	0.813	0.970	0.922	0.648
	MMGDI	0.969	0.967	0.966	0.953	0.963	0.964	0.837
	SWDGDI	0.946	0.953	0.975	0.865	0.970	0.942	0.728
	MDGDI	0.985	0.990	0.989	0.977	0.988	0.986	0.938
HyD-norm-0.5	GDI	0.893	0.872	0.948	0.849	0.926	0.897	0.593
	MMGDI	0.958	0.970	0.975	0.971	0.973	0.970	0.859
	SWDGDI	0.935	0.947	0.964	0.940	0.963	0.950	0.764
	MDGDI	0.988	0.993	0.990	0.989	0.991	0.990	0.953
HyD-norm-0.8	GDI	0.846	0.873	0.950	0.938	0.912	0.904	0.642
	MMGDI	0.968	0.963	0.976	0.978	0.972	0.971	0.864
	SWDGDI	0.949	0.930	0.975	0.971	0.965	0.958	0.803
	MDGDI	0.984	0.993	0.995	0.997	0.994	0.993	0.964

### Study II

Study II evaluated the performance of MLGDI, which had the item exposure control mechanism and was based on MDGDI, against competing item selection strategies. The results of Study 1 indicated that when the test termination rule is reached, MDGDI has the highest classification accuracy compared with MGDI, MMGDI, and CP_SWGDI. MLGDI is a new item selection method based on MDGDI with an additional exposure control mechanism, whereas CP_SWGDI is a new item selection method based on SWGDI with an additional exposure control mechanism. We expect MLGDI to have the highest attribute classification accuracy in MGDI, CP_SWGDI, and MLGDI when the test satisfies the test termination rule.

Study II was conducted to investigate the performance of MLGDI against CP_SWDGDI and GDI. The data generation and evaluation criteria are the same as study I.

#### Results

Table 4 lists the estimates of ACCR, AAMR, and PCCR in each condition. The MLGDI stands out in both the recovery rate of each attribute and the entire profile, followed by CP_SWDGDI. As evident in Table 4, compared with the PCCR of CP_SWDGDI (which also includes an exposure control mechanism), the PCCR of MLGDI increased by approximately 0.15–0.30. Table 4 also indicates that when the test sample reached the test termination condition, MLGDI exhibited the highest accuracy in attribute classification.

**TABLE 4:** Accuracy of the attribute classification for five attributes.

	Item selection method	Attribute (ACCR)					AAMR	PCCR
		A1	A2	A3	A4	A5		
HD-unif	GDI	0.910	0.814	0.975	0.949	0.956	0.921	0.642
	CP_SWDGDI	0.931	0.924	0.937	0.943	0.926	0.932	0.709
	MLGDI	0.981	0.973	0.986	0.987	0.971	0.979	0.911
HD-norm-0.5	GDI	0.946	0.854	0.856	0.901	0.942	0.900	0.598
	CP_SWDGDI	0.910	0.931	0.930	0.931	0.929	0.926	0.676
	MLGDI	0.985	0.984	0.988	0.988	0.993	0.988	0.945
HD-norm-0.8	GDI	0.833	0.871	0.882	0.939	0.907	0.886	0.592
	CP_SWDGDI	0.924	0.918	0.938	0.932	0.931	0.929	0.681
	MLGDI	0.987	0.988	0.986	0.989	0.988	0.988	0.944
LD-unif	GDI	0.973	0.852	0.982	0.913	0.821	0.908	0.599
	CP_SWDGDI	0.903	0.914	0.919	0.906	0.903	0.909	0.643
	MLGDI	0.956	0.949	0.967	0.939	0.944	0.951	0.811
LD-norm-0.5	GDI	0.866	0.914	0.914	0.950	0.793	0.887	0.541
	CP_SWDGDI	0.895	0.903	0.904	0.900	0.886	0.898	0.580
	MLGDI	0.968	0.976	0.963	0.973	0.952	0.966	0.859
LD-norm-0.8	GDI	0.912	0.866	0.885	0.913	0.935	0.902	0.595
	CP_SWDGDI	0.890	0.879	0.897	0.902	0.908	0.895	0.555
	MLGDI	0.963	0.960	0.969	0.961	0.974	0.965	0.850
HyD-unif	GDI	0.937	0.908	0.983	0.813	0.970	0.922	0.648
	CP_SWDGDI	0.923	0.918	0.927	0.913	0.912	0.919	0.658
	MLGDI	0.973	0.978	0.980	0.964	0.971	0.973	0.885
HyD-norm-0.5	GDI	0.893	0.872	0.948	0.849	0.926	0.897	0.593
	CP_SWDGDI	0.914	0.911	0.927	0.926	0.924	0.921	0.653
	MLGDI	0.982	0.977	0.991	0.984	0.986	0.984	0.929
HyD-norm-0.8	GDI	0.846	0.873	0.950	0.938	0.912	0.904	0.642
	CP_SWDGDI	0.903	0.912	0.930	0.919	0.919	0.917	0.642
	MLGDI	0.978	0.986	0.990	0.988	0.988	0.986	0.934

### MLGDI Can Reduce the Participant’s Exposure Rate of MDGDI and Yield a High Accuracy in the Attribute Classification

Table 5 presents the exposure indicators of each item under the different examinee populations, item pools, and six item strategies (MGDI, MMGDI, SWDGDI, CP_SWDGDI, MDGDI, and MLGDI). It is worth noting that, as the exposure control index was added to the MLGDI, the decrease in PCCR was relatively small compared to MDGDI which has the highest PCCR comparing to the other selection item methods, but result in a better item bank usage. As detailed in Table 5, the chi-square value of the item-bank exposure rate of the four item selection strategies without exposure restriction exceeded 110, the TOR exceeded 0.15, and the maximum item exposure rate reached >0.50. Although the accuracy of the MDGDI’s attribute classification was the highest among the six item strategies, the exposure rate of the relevant item bank was also higher than those of the other five strategies. For example, LD-norm-0.5, the chi-square value of MDGDI’s exposure rate was as high as 250, the TOR was as high as 0.349, and the maximum exposure rate of the title was as high as 0.753. MLGDI was the selected item strategy that integrated exposure inhibition based on MDGDI. As indicated in Table 5, MLGDI could effectively reduce the exposure index of each item while considering the high accuracy of attribute classification. Similarly, the chi-square value of MLGDI’s item exposure rate was 75, which was 175 less than that of MDGDI. The TOR of MLGDI was 0.122, which was less than that of MDGDI by 0.227. The maximum exposure rate of the MLGDI’s item was 0.190, which was 0.563 lower than that of MDGDI. With respect to the mastery pattern correct classification rate, the PCCR value of MDGDI was 0.959, and that of MLGDI was 0.945. The mastery pattern correct classification rate decreased by 0.014, and the attribute classification accuracy remained high. In addition, the MLGDI’s mastery pattern correct classification rate remained higher than those of MGDI, MMGDI, SWDGDI, and CP_SWDGDI, and its mastery pattern correct classification rate was second only to that of MDGDI. Table 5 also indicates that although CP_SWDGDI had the highest performance in each index of item exposure rate, the excessive exposure inhibition component added by CP_SWDGDI resulted in a low item selection efficiency and a low accuracy in attribute classification. In the case of LD-norm-0.5, the MLGDI’s PCCR value was 0.945, but that of CP_SWDGDI’s was only 0.676, which was less than that of MLGDI by 0.269. Therefore, although CP_SWDGDI can reduce the items exposure rate, it has a low item selection efficiency and a low accuracy in attribute classification. Therefore, CP_SWDGDI cannot execute a desirable exposure control while maintaining a relatively high classification accuracy in the item selection test.

**TABLE 5:** Item exposure of each item selection method for five attributes.

	Item selection method	Index			
		χ^2^	TOR	max_expose	PCCR
HD-unif	GDI	125.444	0.187	0.764	0.642
	MMGDI	302.007	0.415	1.000	0.853
	SWDGDI	121.937	0.183	0.566	0.688
	MDGDI	326.991	0.448	0.936	0.953
	CP_SWDGDI	0.025	0.026	0.030	0.709
	MLGDI	75.726	0.123	0.190	0.911
HD-norm-0.5	GDI	112.473	0.171	0.574	0.598
	MMGDI	263.488	0.366	1.000	0.887
	SWDGDI	139.819	0.206	0.433	0.778
	MDGDI	250.257	0.349	0.753	0.959
	CP_SWDGDI	0.031	0.026	0.032	0.676
	MLGDI	75.008	0.122	0.190	0.945
HD-norm-0.8	GDI	122.464	0.184	0.458	0.592
	MMGDI	253.052	0.352	1.000	0.864
	SWDGDI	181.618	0.260	0.488	0.800
	MDGDI	264.183	0.366	0.660	0.962
	CP_SWDGDI	0.038	0.026	0.032	0.681
	MLGDI	78.197	0.126	0.190	0.944
LD-unif	GDI	159.785	0.232	0.786	0.599
	MMGDI	297.878	0.410	1.000	0.769
	SWDGDI	136.547	0.202	0.595	0.728
	MDGDI	291.227	0.401	0.893	0.868
	CP_SWDGDI	0.024	0.026	0.030	0.643
	MLGDI	67.731	0.113	0.190	0.811
LD-norm-0.5	GDI	111.453	0.169	0.533	0.541
	MMGDI	284.401	0.393	1.000	0.815
	SWDGDI	141.231	0.208	0.480	0.715
	MDGDI	235.690	0.330	0.693	0.900
	CP_SWDGDI	0.032	0.026	0.031	0.580
	MLGDI	72.465	0.119	0.190	0.859
LD-norm-0.8	GDI	146.151	0.214	0.488	0.595
	MMGDI	270.456	0.375	1.000	0.819
	SWDGDI	179.267	0.257	0.491	0.709
	MDGDI	253.893	0.353	0.642	0.877
	CP_SWDGDI	0.036	0.026	0.033	0.555
	MLGDI	79.315	0.128	0.190	0.850
HyD-unif	GDI	178.761	0.256	0.843	0.648
	MMGDI	250.533	0.349	1.000	0.837
	SWDGDI	148.291	0.217	0.729	0.728
	MDGDI	301.685	0.415	0.918	0.938
	CP_SWDGDI	0.029	0.026	0.030	0.658
	MLGDI	72.495	0.119	0.190	0.885
HyD-norm-0.5	GDI	128.105	0.191	0.567	0.593
	MMGDI	260.627	0.362	1.000	0.859
	SWDGDI	151.950	0.222	0.429	0.764
	MDGDI	263.093	0.365	0.691	0.953
	CP_SWDGDI	0.038	0.026	0.032	0.653
	MLGDI	80.168	0.129	0.190	0.929
HyD-norm-0.8	GDI	151.469	0.221	0.525	0.642
	MMGDI	286.640	0.395	1.000	0.864
	SWDGDI	197.969	0.281	0.480	0.803
	MDGDI	274.848	0.380	0.682	0.964
	CP_SWDGDI	0.037	0.026	0.032	0.642
	MLGDI	79.190	0.128	0.190	0.934

Table 6 shows the percentage of tests that met the attribute-coverage requirement, both at the attribute and overall test levels. For instance, the first entry in the table is 0.675, meaning 67.5% of the tests under the GDI method met the coverage constraint of the first attribute, or that 67.5% of the tests had at least three items measuring the first attribute. Compared with the uncontrolled method, MMGDI, SWDGDI, MDGDI, MLGDI, and CP_SWDGDI produced noticeably better results in balancing the attribute coverage: 100% of the tests met all the attribute coverage requirements. This was more pronounced at the overall test level: with the GDI method, only approximately 10–54% of the tests had an adequate attribute coverage among the conditions, whereas the other three methods ensured that every test is so.

**TABLE 6:** Attribute coverage balance of each item selection method under five attributes.

	Item selection method	Attribute coverage balance					Total balance
		A1	A2	A3	A4	A5	
HD-unif	GDI	0.675	0.395	0.761	0.699	0.708	0.118
	MMGDI	1.000	1.000	1.000	1.000	1.000	1.000
	SWDGDI	1.000	1.000	1.000	1.000	1.000	1.000
	MDGDI	1.000	1.000	1.000	1.000	1.000	1.000
	CP_SWDGDI	1.000	1.000	1.000	1.000	1.000	0.999
	MLGDI	1.000	1.000	1.000	1.000	1.000	1.000
HD-norm-0.5	GDI	0.883	0.607	0.697	0.739	0.747	0.276
	MMGDI	1.000	1.000	1.000	1.000	1.000	1.000
	SWDGDI	1.000	1.000	1.000	1.000	1.000	1.000
	MDGDI	1.000	1.000	1.000	1.000	1.000	1.000
	CP_SWDGDI	1.000	1.000	1.000	0.999	1.000	0.999
	MLGDI	1.000	1.000	1.000	1.000	1.000	1.000
HD-norm-0.8	GDI	0.776	0.810	0.833	0.890	0.691	0.403
	MMGDI	1.000	1.000	1.000	1.000	1.000	1.000
	SWDGDI	1.000	1.000	1.000	1.000	1.000	1.000
	MDGDI	1.000	1.000	1.000	1.000	1.000	1.000
	CP_SWDGDI	1.000	1.000	1.000	1.000	1.000	1.000
	MLGDI	1.000	1.000	1.000	1.000	1.000	1.000
LD-unif	GDI	0.948	0.638	0.952	0.585	0.411	0.103
	MMGDI	1.000	1.000	1.000	1.000	1.000	1.000
	SWDGDI	1.000	1.000	1.000	1.000	1.000	1.000
	MDGDI	1.000	1.000	1.000	1.000	1.000	1.000
	CP_SWDGDI	1.000	1.000	1.000	1.000	1.000	1.000
	MLGDI	1.000	1.000	1.000	1.000	1.000	1.000
LD-norm-0.5	GDI	0.763	0.844	0.880	0.918	0.466	0.241
	MMGDI	1.000	1.000	1.000	1.000	1.000	1.000
	SWDGDI	1.000	1.000	1.000	1.000	1.000	1.000
	MDGDI	1.000	1.000	1.000	1.000	1.000	1.000
	CP_SWDGDI	1.000	1.000	1.000	1.000	1.000	0.999
	MLGDI	1.000	1.000	1.000	1.000	1.000	1.000
LD-norm-0.8	GDI	0.903	0.820	0.830	0.828	0.898	0.481
	MMGDI	1.000	1.000	1.000	1.000	1.000	1.000
	SWDGDI	1.000	1.000	1.000	1.000	1.000	1.000
	MDGDI	1.000	1.000	1.000	1.000	1.000	1.000
	CP_SWDGDI	1.000	1.000	1.000	1.000	0.999	0.999
	MLGDI	1.000	1.000	1.000	1.000	1.000	1.000
HyD-unif	GDI	0.699	0.671	0.928	0.382	0.860	0.125
	MMGDI	1.000	1.000	1.000	1.000	1.000	1.000
	SWDGDI	1.000	1.000	1.000	1.000	1.000	1.000
	MDGDI	1.000	1.000	1.000	1.000	1.000	1.000
	CP_SWDGDI	1.000	1.000	1.000	1.000	1.000	0.999
	MLGDI	1.000	1.000	1.000	1.000	1.000	1.000
HyD-norm-0.5	GDI	0.785	0.781	0.926	0.586	0.718	0.275
	MMGDI	1.000	1.000	1.000	1.000	1.000	1.000
	SWDGDI	1.000	1.000	1.000	1.000	1.000	1.000
	MDGDI	1.000	1.000	1.000	1.000	1.000	1.000
	CP_SWDGDI	1.000	1.000	1.000	1.000	1.000	1.000
	MLGDI	1.000	1.000	1.000	1.000	1.000	1.000
HyD-norm-0.8	GDI	0.818	0.789	0.898	0.900	0.815	0.542
	MMGDI	1.000	1.000	1.000	1.000	1.000	1.000
	SWDGDI	1.000	1.000	1.000	1.000	1.000	1.000
	MDGDI	1.000	1.000	1.000	1.000	1.000	1.000
	CP_SWDGDI	1.000	1.000	1.000	0.999	1.000	0.999
	MLGDI	1.000	1.000	1.000	1.000	1.000	1.000

As shown in Table 6, both the MDGDI and MLGDI methods yielded a perfect attribute balancing, with 100% of the tests under all the conditions fulfilling all attribute coverage, or 100% of these tests having three or more items measuring each of the five attributes.

In addition, the ABI of MDGDI and MLGDI incorporates the dynamic balance of test attributes. Consequently, in the entire test process, the measurement frequency of all attributes is relatively balanced; that is, the difference between the maximum and minimum number of attribute measurements are kept within a given range, which increases the item selection efficiency. Therefore, MDGDI and MLGDI have a higher attribute classification accuracy than do MGDI, MMGDI, SWDGDI, and CP_SWDGDI.

## Discussion and Conclusion

Cognitive diagnostic CAT captures the advantages of both CDA and CAT, allowing the individualized diagnostic feedback with fewer items. In this article, two new item selection methods, the MLGDI and MDGDI, were introduced, and their efficiency were compared with the existing methods. The results indicated that the MDGDI method successfully balanced the attribute coverage in CD-CAT and the MLGDI method simultaneously achieved balance over the attribute coverage and ensured the test security.

Both the MDGDI and the MLGDI outperformed the GDI, MMGDI, SWDGDI, and CP_SWDGDI in terms of the classification accuracy. Compared with MDGDI, MLGDI provides a better item exposure control. The studies also showed that items with HD or high correlations among attributes provided better classification rates.

### MDGDI and MLGDI Have Higher Pattern Determination Rates

The study demonstrated that MDGDI and MLGDI had a higher attribute correct classification rate than GDI, MMGDI, SWDGDI, and CP_SWDGDI under the different conditions. The PCCRs of MMGDI, SWDGDI, and CP_SWDGDI were worse than those of MDGDI and MLGDI. This was attributable to the multiplicative form of the attribute balance indicator in the MMGDI (Cheng, 2010). In such a form, negative–negative–positive cases can occur, which reduces the item selection efficiency of MMGDI. In addition, in the process of the prophase research item, because of the ABI, SWDGDI and CP_SWDGDI (Lin and Chang, 2018) attribute the propensity to choose more items. Specifically, Lin and Chang (2018) found that compared with the simple *q* vector (i.e., a vector with less or a single measurement attribute), an excessively complex *q* vector (i.e., one with multiple measured attributes) reduces the classification accuracy of the measurement (Madison and Bradshaw, 2015; Huebner et al., 2018). The MD index was adopted in MDGDI and MLGDI to achieve attribute coverage balance. Consequently, the measurement drying of all attributes are relatively balanced in the whole test process, that is, the deviation between the minimum and maximum number of attributes measured is within a given range. Therefore, the attributes of MDGDI and MLGDI are more balanced in the test process, which reduces the interference of the original selection strategy of the index and disallows the selection strategy from being more inclined to select some types of items due to the addition of the ABI. Therefore, MDGDI and MLGDI have a higher attribute classification accuracy.

### MLGDI Provides a Better Exposure Control and High Attribute Classification Accuracy

Studies have shown that MDGDI has the highest attribute correct classification rate among MGDI, MMGDI, SWDGDI, CP_SWDGDI, MDGDI, and MLGDI. However, MDGDI also has problems such as the item overexposure, high TOR, and overuse of some items. Therefore, we added the restricted exposure index to MDGDI to construct the MLGDI item selection method. We found that MLGDI (1) greatly reduced the exposure rate and TOR but still had a high attribute classification accuracy and (2) had a pattern determination rate that was second only to MDGDI. In addition, the selected item strategy of CP_SWDGDI considers both the attribute coverage balance and exposure control. The exposure control method of CP_SWDGDI contains two key parameters, namely the adjustment information interval parameter *s* and the exposure parameter *r*, which is the maximum exposure required for a specific test purpose. Therefore, CP_SWDGDI uses the exposure parameter*r*and the adjustment information interval parameter*s*to control the item exposure. However, determining the value of the information interval parameter*s*that is appropriate for a given test length and number of attributes is difficult, which makes the CP_SWDGDI difficult to apply in practice (Zheng and Wang, 2017). Compared with CP_SWDGDI, MLGDI only realizes the exposure control of the participant through the exposure parameter*r*. The absence of the information interval parameter*s*and the need to determine the appropriate*s*value under the different conditions makes MLGDI more practicable. In addition, MLGDI has a higher attribute classification accuracy than does CP_SWDGDI. In conclusion, MLGDI can better meet the requirements of exposure control and has a high attribute classification accuracy, making MLGDI more suitable for practical applications.

The simulation studies yielded the following conclusions:

(1) When only the accuracy of attribute classification and attribute-coverage requirement are considered, MDGDI had the best attribute classification accuracy among GDI, MMGDI, SWDGDI, and MDGDI.
(2) When the accuracy of attribute classification, attribute-coverage requirement, and control item exposure rate are considered, MLGDI had the best attribute classification accuracy among GDI, CP_SWDGDI, and MLGDI.
(3) Adding a restricted item exposure mechanism to the item selection method will reduce the classification accuracy of the attributes of the item selection method.
(4) Compared with GDI, MMGDI, SWDGDI, CP_SWDGDI, and MDGDI, MLGDI can better achieve the attribute-coverage requirement, control item exposure rate, and attribute correct classification rate.

### Directions for Future Research

Future studies can build upon our analysis of the performance of the six item selection strategies (GDI, MMGDI, SWDGDI, CP_SWDGDI, MDGDI, and MLGDI) under different conditions. (1) In the simulations, we found that MDGDI and MLGDI methods can be well used in the selection of CD-CAT projects. However, simulation results are limited to the given simulation conditions. Therefore, to further demonstrate the effectiveness of our method, future research should involve the practical application of the two proposed methods in the use of CD-CAT item banks. (2) For simplicity, we assumed that the correlations between the attributes were set at 0.5 and 0.8 in our simulations. Future studies can test the effectiveness of our thematic strategies (MDGDI and MLGDI) under more realistic conditions. (3) Future studies can extend the MDI and ML indexes to the method based on the expected Shannon entropy and the method based on the *a posteriori*, weighted KL information.

## Data Availability Statement

The raw data supporting the conclusions of this article will be made available by the authors, without undue reservation.

## Author Contributions

JL and LM proposed the original concept, designed the fundamental study of the manuscript, wrote the simulation study code, and organized the article. All authors contributed to the manuscript revision.

## Conflict of Interest

The authors declare that the research was conducted in the absence of any commercial or financial relationships that could be construed as a potential conflict of interest.

## References

[B1] Chang H. Ying Z. (1996). A global information approach to computerized adaptive testing. *Appl. Psychol. Meas.* 20 213–229. 10.1177/014662169602000303

[B2] Chang H. H. (2015). Psychometrics behind computerized adaptive testing. *Psychometrika* 80 1–20. 10.1007/s11336-014-9401-5 24499939

[B3] Cheng Y. (2009a). *Computerized Adaptive Testing for Cognitive Diagnosis*. ed. Weiss D. J., *Presented at the 2009 GMAC Conference*.

[B4] Cheng Y. (2009b). When cognitive diagnosis meets computerized adaptive testing: CD-CAT. *Psychometrika* 74 619–632. 10.1007/s11336-009-9123-2

[B5] Cheng Y. (2010). Improving cognitive diagnostic computerized adaptive testing by balancing attribute coverage: the modified maximum global discrimination index method. *Educ. Psychol. Meas.* 70 902–913. 10.1177/0013164410366693

[B6] Cheng Y. Chang H. H. (2009). The maximum priority index method for severely constrained item selection in computerized adaptive testing. *Br. J. Math. Stat. Psychol.* 62 369–383. 10.1348/000711008X304376 18534047

[B7] Cover T. M. Thomas J. A. (1991). *Elements of Information Theory.* New York, NY: John Wiley.

[B8] Gierl M. J. Wang C. Zhou J. (2008). Using the attribute hierarchy method to make diagnostic inferences about examinees’ cognitive skills in algebra on the SAT©. *J. Technol. Learn. Assess.* 6 1–53.

[B9] Hartz S. M. (2002). *A Bayesian Framework for the Unified Model for Assessing Cognitive Abilities: Blending Theory with Practicality.* Urbana-Champaign, IL: University of Illinois. unpublished doctoral dissertation.

[B10] Hartz S. M. Roussos L. A. (2008). *The Fusion Model for Skill Diagnosis: Blending Theory with Practicality (Research Report No. RR-08-71).* Princeton, NJ: Educational Testing Service.

[B11] Huebner A. Finkelman M. D. Weissman A. (2018). Factors affecting the classification accuracy and average length of a variable-length cognitive diagnostic computerized test. *J. Comput. Adapt. Test.* 6 1–14. 10.7333/1802-060101

[B12] Kang H. A. Zhang S. S. Chang H. H. (2017). Dual-objective item selection criteria in cognitive diagnostic computerized adaptive testing. *J. Educ. Meas.* 54 165–183. 10.1111/jedm.12139

[B13] Kaplan M. de la Torre J. Ramón Barrada J. (2015). New item selection methods for cognitive diagnosis computerized adaptive testing. *Appl. Psychol. Measurement* 39 167–188. 10.1177/0146621614554650 29881001 PMC5978538

[B14] Lee Y. S. de la Torre J. Park Y. S. (2012). Relationships between cognitive diagnosis, CTT, and IRT indices: an empirical investigation. *Asia Pac. Educ. Rev.* 13 333–345. 10.1007/s12564-011-9196-3

[B15] Leighton J. Gierl M. (eds) (2007). *Cognitive Diagnostic Assessment for Education: Theory and Applications.* Cambridge: Cambridge University Press.

[B16] Lin C. J. Chang H. H. (2018). Item selection criteria with practical constraints in cognitive diagnostic computerized adaptive testing. *Educ. Psychol. Meas.* 79:001316441879063. 10.1177/0013164418790634 PMC6425095 30911196

[B17] Madison M. J. Bradshaw L. P. (2015). The effects of Q-matrix design on classification accuracy in the log-linear cognitive diagnosis model. *Educ. Psychol. Meas.* 75 491–511. 10.1177/0013164414539162 29795830 PMC5965638

[B18] Mao X. Xin T. (2013). The application of the monte carlo approach to cognitive diagnostic computerized adaptive testing with content constraints. *Appl. Psychol. Meas.* 37 482–496. 10.1177/0146621613486015

[B19] McGlohen M. Chang H. H. (2008). Combining computer adaptive testing technology with cognitively diagnostic assessment. *Behav. Res. Methods* 40 808–821. 10.3758/BRM.40.3.808 18697677

[B20] Wang C. Chang H. H. Huebner A. (2011). Restrictive stochastic item selection methods in cognitive diagnostic computerized adaptive testing. *J. Educ. Meas.* 48 255–273. 10.1111/j.1745-3984.2011.00145.x

[B21] Weiss D. J. (1982). Improving measurement quality and efficiency with adaptive testing. *Appl. Psychol. Meas.* 6 473–492. 10.1177/014662168200600408

[B22] Weissman A. (2007). Mutual information item selection in adaptive classification testing. *Educ. Psychol. Meas.* 67 41–58. 10.1177/0013164406288164

[B23] Xu X. Douglas J. (2006). Computerized adaptive testing under nonparametric IRT models. *Psychometrika* 71 121–137. 10.1007/s11336-003-1154-5

[B24] Xu X. L. Chang H. H. Douglas J. (2003). *A Simulation Study to Compare CAT Strategies for Cognitive Diagnosis.* Paper presented at the Annual Meeting of the American Educational Research Association. Chicago, IL.

[B25] Yao L. Boughton K. A. (2007). A multidimensional item response modeling approach for improving subscale profificiency estimation and classifification. *Appl. Psychol. Meas.* 31 83–105. 10.1177/0146621606291559

[B26] Wang Y. Sun X. Chong W. Xin T. (2020). Attribute Discrimination index-based method to balance attribute coverage for short-length cognitive diagnostic computerized adaptive testing. *Front. Psychol.* 11:224. 10.3389/fpsyg.2020.00224 32180747 PMC7059599

[B27] Zheng C. J. Wang C. (2017). Application of binary searching for item exposure control in cognitive diagnostic computerized adaptive testing. *Appl. Psychol. Meas.* 41 561–576. 10.1177/0146621617707509 29881106 PMC5978473

